# The integrin adhesome and control of anti-tumour immunity

**DOI:** 10.1042/BST20240386

**Published:** 2024-12-06

**Authors:** Emily R. Webb, Annabel Black, Nicole D. Barth, Stefan N. Symeonides, Valerie G. Brunton

**Affiliations:** Cancer Research UK Scotland Centre (Edinburgh), Institute of Genetics and Cancer, University of Edinburgh, Crewe Road South, Edinburgh EH4 2XU, U.K.

**Keywords:** anti-tumour immunity, focal adhesion kinase, integrins

## Abstract

It is widely regarded that the anti-tumour immune response drives clearance of tumours and leads to prolonged survival in patients. However, tumours are adept at reprogramming the surrounding microenvironment to an immunosuppressive milieu to prevent successful immune directed killing. Adhesion of cells to the extracellular matrix is essential for regulating cellular processes such as survival, proliferation and migration. This adhesion is largely conducted via integrins and their related intracellular signalling networks. Adhesion proteins such as focal adhesion kinase (FAK) are expressed in both tumour cells and cells of the surrounding microenvironment, and are often dysregulated in cancers. Recent work has demonstrated that adhesion proteins are contributing to regulation of the immunosuppressive microenvironment within tumours, and could provide a new avenue to target in combination with immunotherapies. Here, we provide an overview of the effort being made to elucidate the roles adhesion proteins play in modulating anti-tumour responses within a variety of cancer settings. In particular we focus on the multifaceted role of FAK within the tumour immune microenvironment. Finally, we summarise the data in clinical trials, where targeting FAK is being exploited to prime the tumour microenvironment and create potent responses when combined with immunotherapies.

## Introduction

The interplay between the tumour microenvironment (TME) and the immune cells which infiltrate it has now been firmly established as a crucial element in our understanding of how to induce potent anti-tumour immune responses resulting in sustained survival. The tumour immune microenvironment (TIME) varies between different cancer types but consists of several immune cell types which either support tumour immunosuppression and growth, or work to illicit a potent anti-tumour immune response. Immune cells such as T-regulatory cells (Treg), macrophages and neutrophils are largely co-opted by the tumour to perform pro-tumourigenic functions and help tumour cells evade immune cell killing by numerous mechanisms [1–3]. Whereas, natural killer (NK) cells, dendritic cells (DCs), cytotoxic (CD8^+^) and helper (CD4^+^) T cells are considered essential for tumour cell killing, and mounting a potent and sustained anti-tumour immune response. However, in most TIMEs, cytotoxic immune responses are dampened by the profoundly immunosuppressive mechanisms enacted by the tumour and its co-operator immune cells. These include down-regulation of the major histocompatibility complex, metabolic imbalances, secretion of suppressive cytokines, up-regulation of suppressive T cell checkpoint ligands and general immune cell exclusion [3]. One method of overcoming these barriers to achieving potent anti-tumour immunity is the use of immunotherapies which can either boost immune responses (e.g. targeting CD40 on DCs) or dampen immunosuppressive mechanisms (e.g. PD-1 or PD-L1 blockade) [3,4]. Although immunotherapy has had remarkable successes in certain cancers such as melanoma and non-small cell lung cancer (NSCLC), its efficacy has been limited in other cancer contexts, largely due to the immunosuppressive nature of the TIME [4]. Therefore, combination strategies which target the underlying suppressive nature of the TIME are currently being developed to effectively prime the microenvironment for immunotherapy.

Here we will review the role of integrin-dependent adhesion signalling in modulating the TIME and in particular focal adhesion kinase (FAK). Small molecule FAK inhibitors are enabling this pathway to be targeted and are being evaluated in combination with immunotherapies in a number of different solid tumour types.

## Integrin-dependent adhesion signalling

**Figure 1.**
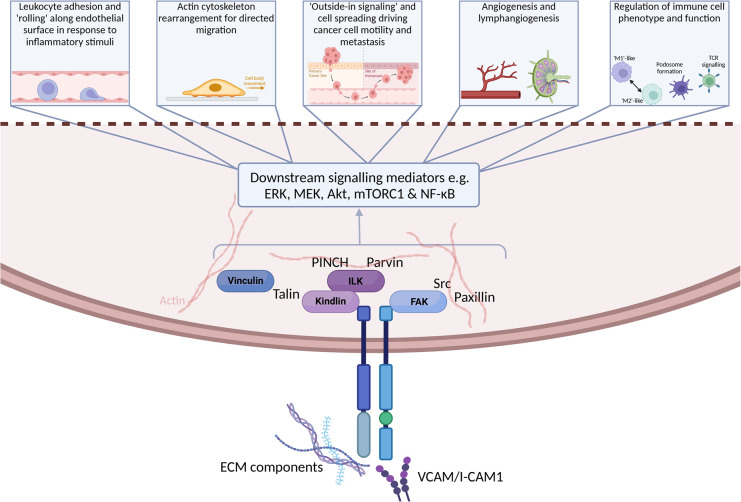
Biological outcomes of integrin-driven adhesion signalling. Binding of integrin ligands, including ECM components (e.g. collagens, proteoglycans, hyaluronan) or adjacent cell surface molecules (e.g. VCAM-1/I-CAMs) results in recruitment of adhesome kinases and adaptor proteins to drive downstream signalling cascades capable of regulating multiple biological processes (light grey boxes, top). Created in BioRender. Black, A. (2024) https://BioRender.com/k82w180.

Cell adhesion to the surrounding extracellular matrix (ECM) is critical for normal tissue development and plays a key role in pathogenesis including cancer development [5]. Integrins are the main cell-ECM adhesion receptors and are composed of 24α and β transmembrane heterodimers made up from 18α and 8β subunits. They act as key signalling hubs known collectively as the integrin adhesome [6,7] where they link both outside-in and inside-out signalling from the ECM to the cellular actomyosin contractility machinery [8–11] (Figure 1). These integrin-dependent adhesions are dynamic structures which control cancer cell behaviours including cell migration, invasion, colonisation at metastatic sites and anchorage-independent survival of circulating tumour cells. The integrin adhesome brings together adaptor proteins, kinases and cytoskeletal proteins which transmit signals from the ECM to the cell interior. These include FAK which is a key signalling molecule within integrin adhesions that initiates downstream signalling events and plays a central role in cell adhesion and migration (Figure 1) [12]. Other components of the integrin adhesome include the adaptor protein talin and the kindlin family of adaptor proteins (Kindlin-1, -2, and -3) which are essential for integrin activation and inside-out signalling [13], while talin and the cytoskeletal protein vinculin play a role in mechanosensing and regulating associations with the actin machinery [6,8]. Integrins are also widely expressed in leukocytes where they are involved in many aspects of the inflammatory response [11,14]. For example, lymphocyte function-associated antigen 1 (LFA-1) αLβ2 is the main integrin expressed on T cells which controls the interaction between T cells and antigen-presenting cells, as well as the migration of T cells to sites of inflammation. A number of the proteins associated with the integrin adhesome are conserved in immune cells but there are some important differences. For example, T cells predominantly express the FAK homolog PYK2 which plays an important role in signalling downstream of β subunit containing integrins and in T cell receptor (TCR) signalling, T cell migration and chemotaxis [15–20]. Unlike kindlin-1 and -2, kindlin-3 has a restricted tissue distribution and is expressed mainly by haematopoietic cells. As kindlins are essential for integrin activation, kindlin-3 plays a key role in regulating integrin-dependent immune cell functions [21–25]. Moreover, mutations in kindlin-3 result in defective integrin activation in leucocytes and platelets and leads to leucocyte adhesion deficiency III in humans [26].

## Adhesion signalling and control of anti-tumour immunity

### Integrins

There is limited information on the direct role of integrins in regulating the TIME. However, αvβ6 and αvβ8 are two of the main regulators of latent TGFβ release and activation [27]. TGFβ is a key regulator of the immune landscape in tumours having widespread effects on the function and differentiation of T cells, as well as inhibiting the function of NK cells, and inhibiting DC, macrophage and neutrophil polarisation and function (reviewed in [27]). Genetic deletion of αv in a melanoma model reduced levels of activated TGFβ and increased intratumoural levels of cytotoxic T cells which was accompanied by prolonged survival following treatment with an anti-PD-1 antibody [28]. Other studies have utilised antibodies to block αvβ6 or αvβ6/8 and shown profound changes in the TIME, and survival advantages in syngeneic murine models when used in combination with anti-PD-1 treatments [29,30]. αvβ8 blocking antibodies can also block tumour growth in syngeneic murine models, which when combined with anti-PD-1 treatment significantly increases survival compared with treatment with either single agent. This was again accompanied by changes in the TIME, including increased infiltration of cytotoxic T cells and pro-inflammatory tumour-associated macrophages (TAMs). The authours suggested that αvβ8 expression on the tumour cells led to localised activation of latent TGFβ presented by immune cells within the TME, which in turn regulated the phenotype of the TIME [31]. β8 is also expressed on Tregs, and in murine models of melanoma and breast cancer, αvβ8 expressing Tregs activated TGFβ that was required to promote anti-tumour immunity, while *ex vivo* treatment of patient samples with anti-β8 antibodies improved cytotoxic T cell activity in tumour explants. In addition to these preclinical studies, data from clinical studies have shown that decreased tumour αv expression correlates with improved survival in NSCLC patients who have been given anti-PD-L1 therapy [28], while increased *ITGB8* on tumour infiltrating lymphocytes (TILs) is associated with poor survival in a number of tumour types [32]. Together these studies support a role for αvβ6 and αvβ8 in regulating the immunosuppressive TIME, and that strategies to block their activity could provide important immunomodulatory functions [32].

β7 is expressed on immune cells where it forms α4β7 and αEβ7 heterodimers which are involved in lymphocyte homing in the gut via binding to cell adhesion molecules (MAdCAM-1 and E-cadherin) within the intestine. In human colorectal cancer (CRC), α4β7 is expressed in a wide range of immune cells while αEβ7 has a more restricted distribution on CD8^+^ T cells and a small subset of NK cells and DCs. Furthermore, expression of the β7 (*ITGB7*) subunit, correlated with increased patient survival, higher cytotoxic immune cell infiltration and better response to immunotherapy [33]. Experiments in *Itgb7^−^*^/*−*^ mice showed increased tumourigenesis in spontaneous and orthotopic murine models of CRC which was associated with reduced migration and homing of T cells into the tumours, although a role for MAdCAM-1 and E-cadherin was not established. When looking specifically at homing of Tregs into a syngeneic model of CRC, β7 was not involved but this required the activity of αLβ2 (LFA-1), and was sufficient for CD8^+^ T cell–mediated tumour regression [34]. Recruitment of macrophages into glioblastoma models has also been shown to be integrin-dependent and mediated via αvβ3 expressed on the infiltrating macrophages [35]. αMβ2 (CD11b/CD18; Mac-1) is expressed on TAMs and subsets of DCs mediating their adhesion to the vasculature and their transendothelial migration. GB1275 is an allosteric modulator of αM that locks αMβ2 in an active conformation and enhances αM mediated adhesion to intercellular adhesion molecule 1 (ICAM-1) on vascular endothelial cells which inhibits transendothelial migration of αM expressing immune cells. In a model of pancreatic cancer, treatment with GB1275 was sufficient to reduce tumour infiltration of CD11b^+^ TAMs, and increase the influx of activated CD103^+^ DCs and CD8^+^ T cells, and combined treatment with GB1275 and an anti-PD-1 blocking antibody gave a significant survival advantage compared with either treatment on its own [36].

### FAK and regulation of the TIME

**Figure 2.**
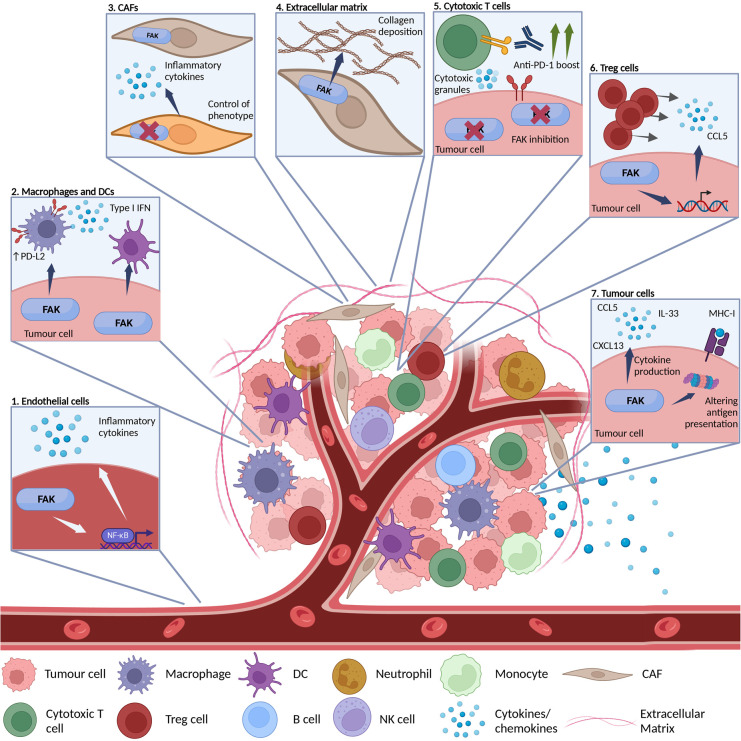
Overview of FAKs multifaceted role in the tumour immune environment. FAK is often overexpressed in tumour cells and also expressed by cells infiltrating the TME such as immune cells and CAFs. Examples of FAKs immune modulation capabilities include: (**1**) FAK expression in endothelial cells can regulate inflammatory cytokine production via NFκB. (**2**) Tumour specific FAK expression controls the phenotype of antigen presenting cells such as macrophages and DCs. (**3**) FAK expression both within tumour cells and CAFs can determine CAF phenotypes and control the inflammatory cytokines they produce. (**4**) FAK expression within stromal cells such as CAFs regulates the ECM (e. g. collagen deposition) thereby regulating the physical barrier to TILs. (**5**) FAK can modulate the effectiveness of immunotherapies targeting cytotoxic T cell populations. (**6**) FAK in tumour cells can translocate to the nucleus and control chemokine transcription, which in turn drives infiltration of suppressive Tregs into the TME. (**7**) Tumour specific FAK expression modulates the secretome by controlling chemokine and cytokine production, alongside regulating antigen processing and presentation via the proteasome. Created in BioRender. Black, A. (2024) https://BioRender.com/k82w180.

The majority of the current research focus on how the integrin adhesome effects anti-tumour immunity has centred around the role FAK plays in modulating adaptive immunity (Table 1, Figure 2). Many cancers are known to dysregulate FAK expression [37] and, in some, FAK expression has been associated with reduced immune infiltrates, for example in pancreatic, ovarian and breast cancer [38–40]. Several studies have now shown that deletion or inhibition of FAK kinase activity can regulate the composition of the TIME by changing the phenotype of multiple immune cell populations thereby fine tuning the anti-tumour immune response and enhancing the efficacy of immunotherapies (Table 1) [38,39,41–46]. For example, in addition to an increase in cytotoxic CD8^+^ T cells, FAK inhibition in pancreatic cancer led to significant reductions in the number of TAMs, Tregs, granulocytes and eosinophils which can all function as immunosuppressive immune cells [38,42]. Furthermore, several studies have demonstrated that deletion or inhibition of FAK can modulate suppressive phenotypes of infiltrating immune cells such as expression of PD-L2 [41,47] and CD206 [38] on TAMs. Single-cell RNA-sequencing analysis of immune cells isolated from FAK inhibitor and radiotherapy treated murine pancreatic tumours showed profound alterations towards anti-tumour phenotypes in numerous populations [42]. This included increases in T cell activation and cytotoxicity, and increased type I interferon production in DCs and TAMs. Recently, it was observed that macrophage specific FAK expression modulates TAM anti-tumour phenotypes downstream of CD11b (αM integrin subunit expressed on myeloid cells) agonism, through a pathway linking STING and interferon signalling [48]. Similar changes in infiltrating immune cell numbers and phenotypes have also been reported in models of squamous cell carcinoma (SCC) and other tumour types [41,44], suggesting that FAK plays a role in modulating anti-tumour immunity in distinct cancer contexts. It has still to be established whether the ability of FAK to regulate the TIME is related to direct effects of FAK in different immune cells such as macrophages and DCs where it is known to control some of their functions e.g. chemotaxis and haptotaxis (Table 1) [49–51].

**Table 1.** Summary of FAKs known effects on the tumour immune microenvironment

Cancer type	Organism	Type of FAK modulation	Effect on immune response	Reference
Role in immune and stromal cells				
PDAC	Human/mouse	Inhibitor (VS-4718)	FAK is activated downstream of αM (CD11b) in TAMs to promote cGAS/STING signalling and enhance anti-tumour CD8+ T cell response via IFNγ production	[48]
PDAC	Human/mouse	Genetic inactivation (kinase dead)	Kinase inactivation in CAFs promotes an M1-like TAM phenotype, modifies tumour ECM and limits spontaneous lung metastasis	[58]
PDAC	Mouse/human	Knockout and inhibitors (GSK2256098, VS-4718 and Defactinib/VS-6063)	FAK controls expression of Psmb8 to regulate antigen processing and presentation, which was independent of FAK kinase activity but dependent on nuclear translocation	[90]
GBM	Human/mouse	Inhibitor (PF-573228)	Inhibition reduces hypoxia-induced HIF-1α and MCT4 to limit monocyte adhesion and GBM cell migration	[92]
Giant cell tumour of bone	Human/mouse	Inhibitor (Defactinib/VS-6063)	FAK is activated via serglycin/CD44 interaction in monocytes and drives differentiation of osteoclasts within tumour to promote disease progression	[93]
GC	Human	N/A	FAK is activated downstream of cyclase-associated protein 2 to drive M2-like macrophage polarisation and formation of a premetastatic niche via TGFβ signalling	[94]
ESCC	Human	Inhibitor (Y15)	FAK is activated in TAMs via tumour-derived IL-32 which promotes M2-like polarisation and lung metastasis	[95]
N/A	Mouse	Inhibitors (PF-573228 and TAE226)	Inhibition causes multinucleation in macrophages and induces a pro-tumoral cytokine profile	[96]
Regulation of cytokines				
PDAC	Mouse	Knockout/Knockdown	FAK controls stromal PD-L2 expression via secretion of IL-6	[47]
SCC	Mouse	Knockout and Inhibitor (VS-4718)	FAK controls Treg numbers in tumours via secretion of CCL5	[44]
SCC	Mouse	Knockout and Inhibitor (VS-4718)	FAK regulates CCL5, IL-33 and sST2 expression	[55]
Glioma	Mouse	N/A	FAK controls macrophage polarisation though CCL5 via LAIR1	[52]
BC	Human	Knockdown	FAK is activated downstream of monocyte secreted CXCL7 to drive invasion and metastasis	[97]
OC	Mouse	Knockout and Inhibitor (VS-4718)	Inhibition increases presence of tertiary lymphoid structures due to secretion of CXCL13, resulting in increased TILs	[39]
Osteosarcoma	Human	Inhibitor (PND-1186/VS-4718)	FAK is activated by IL-8 from oesteosarcoma cells and TAMs to drive proliferation, invasion and lung metastasis	[98]
OSCC	Human	Knockdown	FAK is activated downstream of CCL2/CCR2 in oral CAFs resulting in increased OSCC migration and invasion	[99]
ESCC	Human	Inhibitor (VS-4718) and Knockdown	FAK is activated via TAM-derived CCL22 to activate hedgehog pathway which promotes ESCC stemness and metastasis	[100]
Preclinical studies combining FAK inhibition with immunotherapies				
PDAC	Mouse	Inhibitor (VS-4718)	Reduces numbers of immunosuppressive macrophages, neutrophils and Treg cells, leading to increased response to checkpoint blockade	[38]
PDAC	Mouse	Inhibitor (VS-4718)	When combined with hyaluronic acid degradation, inhibition increases survival with anti-PD-1 therapy, with increases in effector T cells, cytokine modulation and decreased infiltration of CXCR4 expressing myeloid cells	[60]
PDAC	Human/mouse	Inhibitor (VS-4718)	Inhibition rescues negative effects of radiotherapy on the TIME and improves response to anti-PD-1 therapy, with similar changes seen in patient samples	[42]
PDAC/SCC	Mouse	Inhibitor (Ifebemtinib/BI 853520/IN10018)	Inhibition sensitises tumours to anti-4-1BB and anti-OX-40 mAb immunotherapy, due to increasing CD8+ T cell numbers and decreasing suppressive Tregs and macrophages	[41]
BC	Human	Inhibitor (PF-562271)	FAK expression correlates with increased Treg and decreased CD8^+^ T cell signatures, and FAK inhibition in combination with cytokine killer therapy increases survival in xenograft models	[40]
HCC	Mouse	Inhibitor (VS-4718)	Improved survival with combination of FAK inhibition and anti-PD-1 therapy, with decreased Treg and macrophage infiltration	[45]
NSCLC	Mouse	Inhibitor (IN10018)	Inhibition leads to CAF reprogramming and increased T cell infiltration into tumours, leading to improved survival when combined with anti-PD-1 therapy	[57]
Various	Human/mouse	Inhibitor (IN10018)	Combination with pegylated liposome doxorubicin increases survival by boosting immunogenic cell death of tumour cells, and combined synergistically with immune checkpoint therapy	[46]
Various	Mouse	Inhibitor (VS-6063)	Inhibition combined with radiotherapy remodels the tumour stroma leading to increased CD8^+^ T cell infiltration due to changes in ICAM-1 expression	[59]

PDAC, pancreatic ductal adenocarcinoma; BC, breast cancer; SCC, squamous cell carcinoma; GBM, glioblastoma; OC, ovarian cancer; OSCC, oral squamous cell carcinoma; GC, gastric cancer; HCC, hepatocellular carcinoma; ESCC, esophageal squamous cell carcinoma; NSCLC, non-small cell lung cancer.

### FAK and cytokine secretion

Several studies have related changes in the TIME to FAK-dependent regulation of cytokine and chemokine secretion from tumour cells which impact on immune cell infiltration and behaviour (Table 1). For example, in murine models of SCC, FAK drives secretion of the chemokine CCL5 which in turn leads to an increase in Treg accumulation within the TIME [44]. This was dependent on the translocation of FAK to the nucleus in order to control gene transcription. Furthermore, a recent study demonstrated that FAK induced CCL5 secretion was dependent on expression of LAIR1 in glioma cells [52]. Here, LAIR1 allowed FAK to translocate to the nucleus in order to induce CCL5 production, which in turn polarised TAMs within the TIME. Within pancreatic cancer, FAK has been shown to control the expression and secretion of IL-6, which in turn promotes PD-L2 expression on tumour infiltrating antigen presenting cells, possibly leading to a more suppressive TIME [47]. FAKs regulation of another inflammatory cytokine, IL-33, has been well documented in several studies including both inflammation [53] and cancer [52,54,55] contexts. FAK controls chromatin accessibility, downstream IL-33 signalling and expression of IL-33 itself and its soluble receptor sST2. It has recently been reported that FAK plays a role in regulating expression of CXCL13, with inhibition of FAK resulting in the increased presence of tertiary lymphoid structures in ovarian cancer models [39].

### FAK and cross-talk with stromal cells in the TME

FAK can also indirectly regulate the TIME through interactions with other stromal cells in the TME. Cancer associated fibroblasts (CAF) are the main stromal cell population within the TME where they control ECM deposition. They are also closely linked to regulation of the TIME [56]. FAK can influence CAF behaviour and plays a role in stromal reprogramming of tumours [38,42,57–59]. Within murine models of pancreatic cancer, inhibition of FAK resulted in reduced levels of fibrosis due to a reduction in collagen deposition and CAF numbers, leading to decreased immunosuppression [38,42]. Additionally, single cell RNA sequencing analysis of CAFs from pancreatic murine tumours revealed distinct CAF clusters only present in FAK inhibitor treated tumours demonstrating that FAK inhibition can alter CAF phenotypes, including their increased secretion of immunomodulatory cytokines [42,60]. In lung cancer models FAK activity is elevated in CAFs which resulted in increased ECM deposition leading to immune cell exclusion [57]. Addition of a FAK inhibitor normalised the stroma by decreasing CAF dependent collagen deposition.

Another major component of the tumour stroma is the vasculature and FAK is known to play an important role in regulating tumour angiogenesis [12]. Endothelial specific FAK expression can also regulate inflammatory cytokine production, via activation of NF-κB [61–63]. Furthermore, FAK inhibition reduced VCAM-1 expression on endothelial cells which impeded monocyte transmigration [62], with a similar role demonstrated for neutrophil transmigration [64].

Together these data pinpoint FAK as a key regulator of the TIME and demonstrate the pleiotropic role of this adhesion-associated protein in different cellular contexts to generate potent anti-tumour immune responses, both from within cancer cells and other stromal cells in the TME, in addition to systemic immune populations (Figure 2).

### Kindlin and ILK

Many tumour types have dysregulated expression of kindlin-1 and -2 where they are reported to have pro-tumourigenic roles involved with driving more invasive and aggressive disease [26,65]. However, research into the role of kindlins in anti-tumour immunity is in its infancy. We have demonstrated in murine models of breast cancer, that deletion of kindlin-1 results in immune dictated tumour clearance and development of immunological memory [66]. Specifically, kindlin-1 was able to regulate the proportion of Tregs within the TIME, likely by modulating the differentiation of naïve CD4^+^ T cells into Tregs by preventing IL-6 secretion. The link between kindlin-1 expression and IL-6 production has also been observed in humans. Keratinocytes from patients with Kindler syndrome who have loss-of-function mutations in *FERMT1*, the gene that encodes kindlin-1, increase their IL-6 production post-UV exposure [67,68]. However, the exact mechanism by which kindlin-1 can regulate cytokine production is yet to be elucidated. Furthermore, expression of *FERMT1* has been correlated with immune infiltration in pancreatic adenocarcinoma patients [69], supporting a role in influencing adaptive anti-tumour immunity.

Loss of kindlin-2 controls macrophage infiltration and tumour growth in murine models of breast cancer through a mechanism involving a reduction in secretion of the macrophage attractant CSF-1 linked to regulation of TGFβ [70]. In gastric cancer kindlin-2 is also associated with macrophage infiltration where *FERMT2* correlates with levels of M2-like macrophages in human datasets and response rates to immunotherapy [71]. In a model of prostate cancer, deletion of kindlin-3 in T cells and NK cells was reported to increase tumour growth, supporting a role for kindlin-3 in their cytotoxic activity [72]. Analysis of human glioblastoma datasets revealed a positive correlation between *FERMT3* and the infiltration of several immune cells, and a better response to anti-PD-1 therapy. Data from single-cell RNA-sequencing revealed that *FERMT3* was largely expressed in microglial cells and tissue-resident macrophages within the glioblastoma tumours [73].

Analysis of human CRC datasets showed that expression of the kindlin-binding partner integrin linked kinase (ILK) was positively correlated with infiltration of CD8^+^ T cells and immune checkpoint markers, and negatively correlated with B cell infiltration [74]. However, in another CRC study, ILK expression was correlated with infiltration of immunosuppressive Tregs and macrophages [75]. These analyses were compiled from bulk RNA sequencing therefore it is unknown what cells were contributing to ILK expression. More direct evidence that ILK can modulate immune cell function and tumour progression comes from a study in which myeloid specific deletion of ILK reduced tumourigenesis in a spontaneous murine model of CRC which was associated with regulation of macrophage polarisation [76].

## Therapeutic opportunities

### Targeting integrins

Although many integrin targeted therapies have been developed relatively few have been licenced, reflecting the complexity of integrin signalling in different disease contexts [11]. Based on their ability to regulate immune cell recruitment, agents that block α4 function are used for the treatment of inflammatory bowel disease including Crohn's disease and ulcerative colitis, and an anti-αvβ6 monoclonal antibody has been trialled in patients with idiopathic pulmonary fibrosis [77]. However, they are not currently used in oncology, although a number of clinical trials have been undertaken to assess their efficacy in combination with standard of care treatment regimens [78]. Two clinical studies have been reported where targeting integrins has been combined with checkpoint inhibitors (Table 2). Targeting αMβ2 (CD11b/CD18; Mac-1) integrin had limited success in clinical trials which was attributed to incomplete blockade, but based on promising preclinical studies GB1275, an allosteric regulator of the αM (CD11b) subunit which stabilises αMβ2 in an activate state, was trialled in combination with pembrolizumab [36]. However, the trial was terminated due to a lack of benefit either as monotherapy or in combination with pembrolizumab. Currently a phase I dose finding trial is underway with the αvβ8 antagonist (PF 06940434) in combination with anti-PD-1 therapy, which will begin to shed light on the potential for targeting this pathway. As αvβ8 plays an important role in controlling inflammation associated with the development of fibrosis [79,80] through activation of TGFβ, it will be important to understand how to best target αvβ8 in the TME as systemic blocking of TGFβ activation may have unwanted effects on autoimmunity [27].

**Table 2.** Clinical trials targeting adhesion-associated proteins in combination with immunotherapy

Combination	Target	Cancer subtypes	Trial phase	Recruiting/ongoing/completed	Trial number	Reference
**FAK inhibitor**						
**IN10018 (BI-853520)**						
Pegylated liposomal doxorubicin + Toripalimab	Chemotherapy, PD-1	Previously-treated locally advanced or metastatic solid tumour including metastatic TNBC, head and neck squamous cell cancer, platinum-resistant ovarian cancer, small cell lung cancer	Ib/II	Active, not recruiting	NCT05830539	[101]
Nab-paclitaxel + Tislelizumab	Chemotherapy, PD-1/PD-L1	Previously-treated NSCLC	Ib/II	Not yet recruiting	NCT05982522	
Carboplatin + etoposide + Tislelizumab	Chemotherapy, PD-1/PD-L1	Extensive-stage small cell lung cancer (ES-SCLC)	Ib/II	Recruiting	NCT06030258	
Cobimetinib + Atezolizumab	MEK, PD-L1	Metastatic uveal melanoma (UM), NRAS-mutant metastatic melanoma	I	Recruiting	NCT04109456	
**Defactinib (VS-6063)**						
Gemcitabine + Pembrolizumab	Chemotherapy, PD-1	Solid tumours/advanced solid tumours Intractable pancreatic cancer	I	Completed	NCT02546531	[85]
		Pancreatic ductal adenocarcinoma	II	Recruiting	NCT03727880	[102]
Pembrolizumab	PD-1	NSCLC, mesothelioma, pancreatic neoplasms	I/IIA	Unknown	NCT02758587	
		Malignant pleural mesothelioma	Ia-Ib	Withdrawn	NCT04201145	
Avutometinib + Nivolumab	Raf, PD-1	Refractory LKB1-mutant advanced non-small cell lung cancer	II	Recruiting	NCT06495125	
Avelumab	PD-L1	Epithelial ovarian cancer	I/Ib	Terminated	NCT02943317	
**Integrin modulators**						
PF-06940434 + PF-06801591	αvβ8 integrin, PD-1	CCHN (squamous cell carcinoma of the head and neck), renal cell carcinoma (RCC — clear cell and papillary), ovarian, gastric, oesophageal, lung squamous cell, pancreatic and biliary duct, endometrial, melanoma and urothelial tumours	I	Active, not recruiting	NCT04152018	
GB1275 + pembrolizumab	Allosteric modulator αM integrin subunit (CD11b), PD-1	Advanced solid tumours	I	Terminated	NCT04060342	[36]

### Targeting FAK

Another approach has been the use of small molecule inhibitors of FAK, with several in clinical evaluation across a number of tumour types [12,37,81]. Early clinical studies showed that they were well tolerated but had limited activity when used as monotherapy and trials are now evaluating their activity in combination with chemotherapy and targeted therapies, most notably MEK inhibitors [12,37]. However, the demonstration in preclinical studies that FAK inhibition can enhance the efficacy of immune checkpoint blockade (Table 1) opened up the possibility of using FAK inhibitors in combination with immunotherapies [82–84]. This led to rapid translation to clinical studies, and a number of trials are now combining FAK inhibitors with chemo-and or immunotherapy (Table 2). One phase 1 study in advanced refractory pancreatic cancer has reported on the combination of pembrolizumab with gemcitabine and the FAK inhibitor defactinib (VS-6063) [85]. The combination was well-tolerated in patients with no dose-limiting toxicities. Although the number of patients treated in this dose escalation study was small, there were indications of activity for patients who had progressed on prior treatments, as well as for those being given maintenance treatment. There was an encouraging rate of stable disease at first scan (70–80%), with some maintained out to 6 or 12 months, and there was one formal partial response in each group. Encouragingly, analysis of tumour samples pre-and post-treatment showed that in most cases there was an increase in CD8^+^ cytotoxic T cells and effector T cells, with a down-regulation of suppressive TAMs and Tregs, and results of ongoing Phase II studies are eagerly anticipated.

The majority of the commonly used inhibitors target the ATP binding domain and due to the high homology between PYK2 and FAK in the kinase domain (60%) [86], most inhibitors, including defactinib [85], also inhibit PYK2. However, there are also FAK-selective inhibitors including ifebemtinib (IN10018/BI 853520) and narmafotinib (AMP945) in clinical development [87,88]. Given the widespread expression of PYK2 in haematopoietic cells selectivity may modulate the effect and toxicity profile. However, with FAK and PYK2 reported to have both redundant and specific functions across multiple cell types [12], it is not yet known whether FAK-selective or dual FAK/PYK2 inhibition will be most effective in driving anti-tumour immunity in the clinic. Preclinical studies with IN10018 have shown that inhibition of FAK alone is sufficient to increase TILs [41,43,46] and enhance anti-tumour immunity in syngeneic mouse models [41,46].

Despite both promising preclinical studies and encouraging signs of clinical activity, further work is required to investigate the most effective combinations of FAK inhibitors with different immunotherapies, and how to select patients who are most likely to benefit. Efforts towards this have identified regulation of CD155, a checkpoint ligand for TIGIT, by FAK, and FAK expression is positively associated with TIGIT checkpoint ligands in human high grade serous ovarian cancer [39]. Combination of FAK inhibition with a TIGIT blocking antibody prolonged survival in mouse models and was accompanied by increased TILs and reduced TIGIT^+^ T regulatory cells [39]. Another study showed that expression of the T-cell co-stimulatory ligand CD80 on cancer cells sensitises tumours to FAK inhibition, and that in the absence of CD80, targeting alternative T-cell co-stimulatory receptors OX-40 and 4-1BB in combination with FAK, can drive enhanced anti-tumour immunity and even complete regression in syngeneic mouse models [41].

Both kinase-dependent and -independent roles for FAK have been identified with the FAK FERM domain providing protein-protein scaffolding functions that can drive important cancer cell behaviours [37,89]. For example, the FAK-dependent control of antigen processing and presentation has been shown to be via kinase-independent regulation of the immunoproteosome subunit Psmb8 [90]. The use of FAK proteolysis targeting chimera (PROTAC) degraders which inhibit both the scaffolding and kinase functions of FAK [12] will help unravel the complex mechanisms involved in FAK-dependent regulation of the TIME. However, further work is required to optimise their use and determine whether they can be used in a clinical setting.

Earlier lines of therapy and stages of disease, including adjuvant settings, have been the direction of travel for immunotherapies and the role of FAK across the time-course of disease should be considered alongside its site and biology. Optimal combinations may also involve scheduling considerations with other immunotherapies, as well as with the standard of care therapies used in that setting. Key to answering these questions will be ongoing preclinical work in increasingly representative models as well as biomarker-rich clinical trials that establish the biology of FAK inhibition across different TIMEs, and its interaction with other immune pathways.

## Perspectives

- Immunotherapies that activate the immune system have shown remarkable results for many patients. However, for many there is no benefit. Resistance to immunotherapies is multifaceted and presents a real challenge clinically [91]. One approach has been to combine immunotherapies with agents that can modulate the immunosuppressive TIME.
- Integrins are important for controlling the activity of a range of immune cell populations through their ability to regulate adhesion and migration. However, more recently significant advances have been made in understanding how integrin adhesion-associated proteins can regulate the TIME through additional mechanisms including integrin-dependent regulation of immune cell migration and regulation of TGFβ. FAK has emerged as an important regulator that can impact on the TIME through multiple mechanisms and enhance anti-tumour immunity.
- FAK inhibition is being explored in a number of clinical settings, and early-phase trials of cancer immunotherapy combinations are beginning to show encouraging indications of activity. However, the optimal combination partners, population and line of therapy are still to be determined as we tease out the biology of FAK's interaction with other tumour immunology pathways and seek selection biomarkers for TIMEs that are driven by FAK.

## Open Access

Open access for this article was enabled by the participation of University of Edinburgh in an all-inclusive *Read & Publish* agreement with Portland Press and the Biochemical Society under a transformative agreement with JISC.

## Abbreviations

CAF: cancer associated fibroblast
CRC: colorectal cancer
DC: dendritic cell
ECM: extracellular matrix
FAK: focal adhesion kinase
ILK: integrin linked kinase
LFA-1: lymphocyte function associated antigen 1
NK: natural killer
PROTAC: proteolysis targeting chimera
SCC: squamous cell carcinoma
TAM: tumour-associated macrophage
TCR: T cell receptor
TIGIT: T cell immunoreceptor with immunoglobulin and immunoreceptor tyrosine-based inhibitory motif domains
TIL: tumour infiltrating lymphocyte
TIME: tumour immune microenvironment
TME: tumour microenvironment
Treg: T-regulatory cell
